# The exceptionally efficient quorum quenching enzyme LrsL suppresses *Pseudomonas aeruginosa* biofilm production

**DOI:** 10.3389/fmicb.2022.977673

**Published:** 2022-08-22

**Authors:** Zahid Ur Rehman, Afaque A. Momin, Abdullah Aldehaiman, Tayyaba Irum, Raik Grünberg, Stefan T. Arold

**Affiliations:** ^1^Bioscience Program, Biological and Environmental Science and Engineering Division, King Abdullah University of Science and Technology (KAUST), Thuwal, Saudi Arabia; ^2^Environmental Science Program, Water Desalination and Reuse Center, King Abdullah University of Science and Technology (KAUST), Thuwal, Saudi Arabia; ^3^Computational Biology Research Center, King Abdullah University of Science and Technology (KAUST), Thuwal, Saudi Arabia; ^4^Services Hospital, Services Institute of Medical Sciences, Lahore, Pakistan; ^5^Centre de Biologie Structurale (CBS), INSERM, CNRS, Université de Montpellier, Montpellier, France

**Keywords:** quorum quenching enzymes, biofilm, *Pseudomonas aeruginosa*, acyl-homoserine lactones, virulence factors

## Abstract

Quorum quenching (QQ) is the enzymatic degradation of molecules used by bacteria for synchronizing their behavior within communities. QQ has attracted wide attention due to its potential to inhibit biofilm formation and suppress the production of virulence factors. Through its capacity to limit biofouling and infections, QQ has applications in water treatment, aquaculture, and healthcare. Several different QQ enzymes have been described; however, they often lack the high stability and catalytic efficiency required for industrial applications. Previously, we identified genes from genome sequences of Red Sea sediment bacteria encoding potential QQ enzymes. In this study, we report that one of them, named LrsL, is a metallo-β-lactamase superfamily QQ enzyme with outstanding catalytic features. X-ray crystallography shows that LrsL is a zinc-binding dimer. LrsL has an unusually hydrophobic substrate binding pocket that can accommodate a broad range of acyl-homoserine lactones (AHLs) with exceptionally high affinity. *In vitro*, LrsL achieves the highest catalytic efficiency reported thus far for any QQ enzyme with a *K_cat_*/*K_M_* of 3 × 10^7^. LrsL effectively inhibited *Pseudomonas aeruginosa* biofilm formation without affecting bacterial growth. Furthermore, LrsL suppressed the production of exopolysaccharides required for biofilm production. These features, and its capacity to regain its function after prolonged heat denaturation, identify LrsL as a robust and unusually efficient QQ enzyme for clinical and industrial applications.

## Introduction

Quorum sensing (QS) is a chemical communication between bacteria that coordinates gene expression at the community level. QS is mediated by a diverse set of small molecules, among which acyl-homoserine lactones (AHLs) are the best-studied (Whiteley et al., 2017). AHLs play an important role in biofilm formation by various bacteria in many different environments (Eberl et al., 1996; Davies et al., 1998; Lee et al., 2018). Biofilms have detrimental consequences in health care and industries, such as water purification, shipping, and aquaculture (Salta et al., 2013; Rehman et al., 2019, 2020, 2021; Rumbaugh and Sauer, 2020). Once formed, biofilms are difficult to remove because of their increased resistance to antimicrobial and other physical and chemical cleaning procedures. Biofilms incur substantial economic costs, and the current biofilm prevention and treatment practices often negatively affect the environment. Therefore, sustainable and eco-friendly measures to mitigate biofilms are required.

The AHL molecules consist of a lactone ring linked to the acyl chain through an amide bond. The length of the acyl chain can vary from 4 to 18 carbon atoms and may carry an oxo or hydroxyl modification at the third carbon atom (Azimi et al., 2020). The QQ enzymes hydrolyzing the lactone ring of AHLs are called lactonases, whereas those cleaving the amide bond are amidases or acylases. Most QQ enzymes discovered so far are lactonases, whereas relatively few acylases have been identified (Dong et al., 2000; Wang et al., 2012; Tang et al., 2015). Lactonases belong to three protein superfamilies: (i) phosphotriesterase-like lactonases (PLL), (ii) paraoxonases, and (iii) metallo-β-lactamases (MBLs; Fetzner, 2015). AiiA was the first AHL lactonase discovered belonging to the MBL superfamily. It possesses a characteristic HXHXDH motif that binds two metal cations (Dong et al., 2000). Subsequently, AHL lactonases have been discovered in diverse bacteria exhibiting dramatic differences in kinetic parameters, stability, and substrate preference (Fetzner, 2015; Bergonzi et al., 2018).

Acyl-homoserine lactone-based QS plays a vital role in the pathogenicity of many gram-negative bacteria. For example, *Pseudomonas aeruginosa*, a human pathogen (Smith et al., 2002); *Erwinia carotovora*, a plant pathogen (Smadja et al., 2004); and *Aeromonas hydrophila*, a fish pathogen (Chu et al., 2013), use AHLs to regulate the production of virulence factors. The diseases caused by these pathogenic bacteria have a devastating influence on health, agriculture, and food security. Therefore, the inhibition of virulence factor production through the degradation of AHLs is an attractive strategy to reduce the pathogenicity of common bacteria. In contrast to bactericidal approaches, this so-called quorum quenching (QQ) was presented as a sustainable method that does not appear to induce resistance in target bacteria (Cegelski et al., 2008). However, these claims have been challenged, and it was suggested that certain QQ agents that enter the cells could exert selective pressure and impart resistance in bacteria (Defoirdt et al., 2010; Garcia-Contreras et al., 2013). It remains unclear whether QQ enzymes that do not enter the bacterial cell can give rise to resistance.

Although diverse chemical compounds exhibit a QS inhibitory effect, applying a QQ enzyme offers certain advantages. The key parameters for applying QQ enzymes as anti-infective agents are their substrate affinity, catalytic efficiency, and stability (Remy et al., 2020). Protein engineering may improve these factors (Billot et al., 2020). However, different applications require different QQ enzymes to suppress communication mediated through specific AHL molecules in specific environments. Given the widespread use of QS in nature and the diversity of potential QQ applications, there is a need to bio-prospect for novel QQ enzymes with a high affinity for specific AHL molecules, better stability, and improved catalytic activity.

Since their discovery, QQ enzymes have stirred much interest due to their potential use in several fields. Biotechnological applications of QQ enzymes can include those in human health, aquaculture, agriculture, biofouling, and biocorrosion (Billot et al., 2020). Successful application of QQ enzymes in industry requires a repertoire of QQ enzymes with varying substrate specificity, affinity, and stability under various conditions. With this purpose, we identified potential QQ enzymes from Red Sea sediment – a harsh and understudied environment (Renn et al., 2021). We gene-synthesized, expressed and purified the first QQ enzyme, LrsL, isolated from the genus *Labrenzia*. The 1.9 Å resolution crystal structure that we determined for LrsL reveals that it has an exceptionally hydrophobic substrate binding pocket, capable of accommodating a broad range of AHL molecules. Functional studies showed that LrsL exhibits the highest catalytic activity reported for any QQ enzyme thus far on a range of AHL molecules (C4-AHL, C6-AHL, and Oxo-C12-AHL). LrsL has the capability of regaining its functional three-dimensional fold after heat denaturation, which may be beneficial for biotech applications.

## Materials and methods

### Identification of LrsL

Previously, we identified seven QQ lactonase genes in Red Sea sediment bacteria genomes (Rehman and Leiknes, 2018). We selected all seven genes for expression in *Escherichia coli* (BL21 DE3) for the initial screening. The protein structures of QQ enzymes were predicted using SWISS-MODEL (Waterhouse et al., 2018). Unmodeled N- and C-terminal amino acids were assumed to be flexible and were removed before gene synthesis. The codons were optimized for expression in *E. coli* using an in-house Python script (Guo et al., 2021). The codon-optimized genes were gene-synthesized into expression plasmid pJEx411c (Guo et al., 2021), with the in-frame addition of a Twin-Strep-tag at the C-terminal, containing kanamycin resistance for selection. The seven plasmids were transformed into competent *E. coli* cells. Successful transformants were selected by plating on Luria–Bertani (LB) agar medium containing 50 μg/ml kanamycin (GoldBio, United States).

### Bioinformatic analyses

Multiple sequence alignments were produced using the online version of MUSCLE (Edgar, 2004) and were edited using Jalview V-2 (Waterhouse et al., 2009). Phylogenetic analysis was performed in MEGA X using the maximum likelihood method with 500 bootstrap values (Kumar et al., 2018). AlphaFold (Jumper et al., 2021) models were calculated using the AlphaFold2_advanced Colab notebook.

### Expression and purification of LrsL

Table 1 lists all strains and plasmids used in this study. An overnight culture of 10 ml of *E. coli* (BL21 DE3) harboring pJEx411c:LrsL plasmid was inoculated into fresh LB (1 l) with kanamycin (50 μg/ml; GoldBio, United States). The culture was incubated at 37°C with shaking at 200 rpm. At an optical density (OD)_600_ of 0.6, the expression of the QQ enzyme was induced using 250 μM isopropyl ß-D-1-thiogalactopyranoside (GoldBio, United States), and 500 μM ZnSO_4_ (Merck, Germany) was added, followed by incubation with shaking at 22°C for another 22 h. Cells were pelleted by centrifugation at 8000 *g* for 10 min at 4°C. The supernatant was discarded, and the cell pellet was stored at −80°C until further processing. The cell pellet was thawed in water at room temperature and resuspended in 5 ml of cold lysis buffer A (50 mM Tris, 200 mM NaCl, 1 mM tris(2-carboxyethyl)phosphine (TCEP), and 250 μM ZnSO_4_, pH 7.8) for each 1 g of pellet and kept on ice. Benzonase (Millipore, United States) was added at 25 units/ml of buffer. Cells were lysed on a cell disruptor (Constant Systems Ltd.). Cell lysates were centrifuged in a prechilled centrifuge at 6,000 *g* for 10 min at 4°C. The supernatant was collected and recentrifuged at 35,000 *g* for 45 min at 4°C, and filtered using a miracloth (Millipore, United States). The LrsL protein was purified on an AKTA pure (GE Healthcare, United States) in the following sequence: Strep-tag affinity purification, ion-exchange, and gel-permeation chromatography. We used the StrepTrap HP (GE Healthcare) column and binding buffer B (50 mM Tris, 200 mM NaCl, and 1 mM TCEP, pH 8) for affinity purification. Elution was performed in a binding buffer containing 2.5 mM desthiobiotin. Fractions were pooled and concentrated to 5 ml using 10 K Amicon Ultra (Millipore) columns (centrifugation at 4000 *g* for 20 min). Concentrated proteins were dialyzed overnight at 4°C against buffer C (50 mM Tris, 1 mM TCEP, and 10 mM NaCl, pH 8) using 14–16 kDa membranes, subjected to the Mono-Q 10/100 Gl column (GE Healthcare) for ion-exchange purification, and recovered using a high salt buffer (50 mM tris, 1 M NaCl, and 1 mM TCEP, pH 8). Finally, the proteins were subjected to gel-filtration on a Superdex75 16/600 column (GE Healthcare) into buffer D (50 mM HEPES, 150 mM NaCl, and 250 μM ZnSO4, pH 7.8). After spin concentration, protein aliquots were flash-frozen in liquid nitrogen and stored at −80°C. The quality, purity and molecular mass of the recombinant proteins were measured using sodium dodecyl sulfate gel electrophoresis (Invitrogen). Protein concentrations were determined by absorbance on a NanoDrop spectrophotometer using sequence-specific extinction coefficients.^1^

**Table 1:** Bacterial strains and plasmids.

Strain or plasmid strains	Description	Source
*E. coli* BL21 DE3	Bacterial strain for high level expression	Invitrogen
*E. coli* BL21 DE3+ pJEx411c:LrsL	BL21 harboring plasmid for expression of LrsL gene	This study
*Pseudomonas aeruginosa* PAO1	Prototrophic wild-type strain	Holloway et al., 1986
*Chromobacterium violaceum* CV026	ATCC 31532 derivative, *cviI*::Tn*5xylE* Km^r^, Sm^r^	McClean et al., 1997
pJEx411c:LrsL	Expression plasmid containing LrsL gene for expression	This study
pJEx411c:QQ0X	Expression plasmid containing QQ gene (01–07) for expression	This study

### Protein crystallization

Purified LrsL protein was used for crystallization and crystals were obtained using the sitting drop vapor diffusion method. LrsL crystals were obtained by equilibrating 300 nl of protein (10 mg/ml in 50 mM HEPES, 150 mM NaCl, and 250 μM ZnSO4, pH 7.8 buffer) with 300 nl of reservoir solution (1.6 M tri-sodium citrate tribasic) at 25°C using the Mosquito pipettor. Crystals were flash-cooled using liquid nitrogen without any cryo-protectant. Diffraction data were collected at Proxima 1 beamline (SOLEIL Synchrotron, France) at 100 K using a DECTRIS EIGER X 16 M detector (Proposal number 20210195). The data was processed in XDS (Kabsch, 2010).

### Protein structure determination

Diffraction data was checked for possible contaminants using ContaMiner (Hungler et al., 2016). Initial phases were calculated using Balbes (Long et al., 2008). Automated rebuilding was performed by Arp wARP (Langer et al., 2008), followed by iterative manual rebuilding using COOT (Emsley et al., 2010) and automated refinement by Refmac (Murshudov et al., 1997). The protein model was evaluated using MolProbity (Davis et al., 2007).

### Circular dichroism

Circular dichroism spectra measurements were performed in triplicates using JASCO spectropolarimeter, with a 1-mm path-length cell. LrsL was measured at 10 μM concentration in 2.5 mM bicine pH 8.3, 150 mM NaCl, and 0.25 mM ZnSO_4_ buffer. The measurement was performed in a temperature gradient starting at 25°C until 120°C. The sample was then cooled down to 25°C and re-measured. CD results were analyzed using CAPITO (Wiedemann et al., 2013).

### *In silico* molecular docking analyses

The crystal structure of AidC, a Dizinc Quorum-Quenching Lactonase, in complex with N-hexnoyl-L-homoserine (PDB ID: 4zo3) and the crystal structure of LrsL from this study were used to perform docking analyses. The ligand structure of C6L homoserine was extracted from PDB ID: 4zo3 using PyMOL.^2^ Flexible docking was performed using AutoDock 4.2 (Goodsell et al., 1996) as described in (Vogeley et al., 2022), except that the size for the gridbox (x, y, z points) were set to 20 × 20 × 20 and centers for the grid were designated at *X* = 123.28, *Y* = 102.8, *Z* = 32.32 dimensions for PDB ID: 4zo3, whereas for LrsL these parameters were set to 20 × 20 × 20 and *X* = 123.16, *Y* = 102.74, *Z* = 32.85 dimensions, respectively. Final docking poses were analyzed using PyMOL.

### Size exclusion chromatography

Size exclusion chromatography combined with multiangle light scattering was performed on a Superdex 200 10/300 column (Cytiva) on an Agilent HPLC setup. 5.5 mg/ml of LrsL were injected in the column and analyzed in 20 mM HEPES, pH 7.5, 200 mM NaCl, 1 mM TCEP, and 0.01% of NaN_3_. The molecular weight estimation and refractive index were calculated using Astra Multiangle Light Scattering software (Wyatt Technology).

### Differential scanning fluorimetry

The experiment was performed as previously described (Momin et al., 2019). Briefly, thermal stability was assessed in 20 mM HEPES, pH 7.5, 200 mM NaCl, and 1 mM TCEP. Fluorescence was measured by heating the LrsL sample from 25°C to 99°C at 0.03°C/s. LrsL was used at a concentration of 10 μM, and fluorescent dye SYPRO Orange was used at a final concentration of 5X.

### Characterization of QQ activity

The QQ activity of purified enzymes was investigated using *Chromobacterium violaceum* CV026 assay as described previously (Rehman and Leiknes, 2018). Briefly, Luria–Bertani (LB) agar plates were overlaid with 5 ml of 1/100th-dilution of an overnight culture of the biosensor strain CV026 mixed with LB soft agar (0.7%). After the soft agar was solidified, wells (6-mm) were created in the medium by using sterile pipette tips. These wells were filled with the LrsL and C6-AHLs mixture and incubated at 30°C for 24 h. C6-AHLs in buffer D and mixed with BSA were used as controls. The appearance of a purple halo around the well indicated intact C6-AHLs. On the other hand, LrsL degraded C6-AHLs, and therefore, the biosensor strain was not activated. Thus, halo formation was not observed. The minimum active concentration of LrsL was determined as the highest dilution that could degrade 10 μM C6-AHLs in 24 h, by preliminary studies and as determined by the *C. violaceum* assay (Mayer et al., 2018).

### Kinetic characterization

Experiments were performed in a 200 ml reaction volume using a 96-well plate with a path length of 5.8 mm to calculate the catalytic parameters. For the AHL hydrolysis experiment, we used cresol purple (Alfa Aesar, United States) as a pH indicator, as previously described (Bergonzi et al., 2016, 2018). The hydrolysis of the lactone ring was monitored at 577 nm in the buffer (2.5 mM Bicine pH 8.3, 150 mM NaCl, 0.25 mM ZnSO_4_, 0.2 mM cresol purple, and 0.5% of DMSO, at 25°C) using different concentrations of enantiomerically pure AHLs ranging from 0 to 1 mM. A specific concentration of enzyme (1, 2, or 3 μM) was tested for each substrate. Each measurement was made in triplicate. The background rate was measured in the absence of the substrate and enzyme. The AHL degradation rates in the absence of the enzyme were measured as the control and found to be similar to the background rates. Catalytic parameters were calculated by fitting the data to the Michaelis–Menten equation using a rectangular hyperbola curve fitting model using SoftMax Pro 7.0.3 (Molecular Devices).

### Biofilm assay

The overnight culture of *P. aeruginosa* was diluted to an OD of 0.05 and incubated with 1.14 μM of LrsL enzymes in round-bottom 96-well plates at 37°C. Biofilms were assayed 24 h after inoculation using the crystal violet staining method, as described previously (Coffey and Anderson, 2014). For the preformed biofilm assay*, P. aeruginosa* biofilm was grown for 24 h in 96-well plates. The culture medium was carefully removed and replenished with fresh LB containing 1.14 μM of LrsL. The plates were further incubated for 8 h at room temperature, and crystal violet staining was performed to quantify the attached biomass. We have used BSA as a negative control for the effects of adding protein to the experiment (Bergonzi et al., 2019). The concentrations of the enzymes were chosen based on our preliminary analysis and were consistent with previous studies (Zhang et al., 2017; Bergonzi et al., 2019).

### Congo red binding assay

Production of exopolysaccharides was assessed using the previously described assay (Spiers et al., 2003). The overnight culture of *P. aeruginosa* PAO1 was inoculated with fresh LB at a 1:100 dilution. The LrsL and bovine serum albumin (BSA) were added to a final concentration of 1.14 μM. The cultures were incubated at 37°C for 72 h without shaking. The biomass was collected by centrifugation at 13,000 *g* for 5 min, and the supernatant was discarded. The pellet was resuspended in 1 ml of 20 μg/ml Congo red (CR) and incubated for 90 min with shaking at room temperature. The biomass and bound CR were sedimented by centrifugation at 16,000 *g* for 5 min. The supernatant was serially diluted, and optical density was measured at 490 nm. The LB media, PAO1 without LrsL, and PAO1 inoculated with BSA served as controls.

### Statistical analyses

All statistical analyses were performed using Excel (v. 16.52 Microsoft) and data were plotted in GraphPad Prism 9.0.^3^

## Results and discussion

### Identification of LrsL as a promising QQ candidate

As the basis for this study, we used all the seven lactonase genes previously identified bioinformatically as QQ genes in genomes of Red Sea isolates (Rehman and Leiknes, 2018). Based on the predicted protein structures of these genes, we designed expression constructs for their catalytic domain (see Methods). In the initial screening of the recombinantly produced proteins, only the QQ gene from *Labrenzia* sp. VG12 (formerly QQ0007_VG12) encoding protein (ASP32504.1) exhibited good expression and degradation of AHLs (Supplementary Figure S1A). Therefore, we proceeded with the expression, purification, and further analyses of this protein, which we named LrsL (L for *Labrenzia*, rs for the Red Sea, and L for lactonase), representing the genus of the host bacteria, source of bacteria, and protein function. In the sequence used for gene synthesis, we removed the codons for the N-terminal 47 residues of LrsL, encompassing the secretion signal sequence and unstructured region. The resulting recombinant LrsL protein consists of 285 residues and has a calculated molecular mass of 30.8 kDa.

### LrsL forms zinc-binding homodimers

The wild-type LrsL protein consists of 331 amino acid residues and carries an N-terminal twin-arginine translocation (TAT) signal sequence with a cleavage site between residue 38 and 39 as determined using SignalP analysis (Supplementary Figure S1B; Petersen et al., 2011). TAT signals generally serve to translocate folded proteins across membranes. To our knowledge, LrsL is the only QQ enzyme discovered to date that carries a TAT signal and is only the second enzyme that is potentially secreted extracellularly, the other one being MomL (Tang et al., 2015). These extracellular QQ enzymes were both discovered in a marine environment.

**Figure 1:**
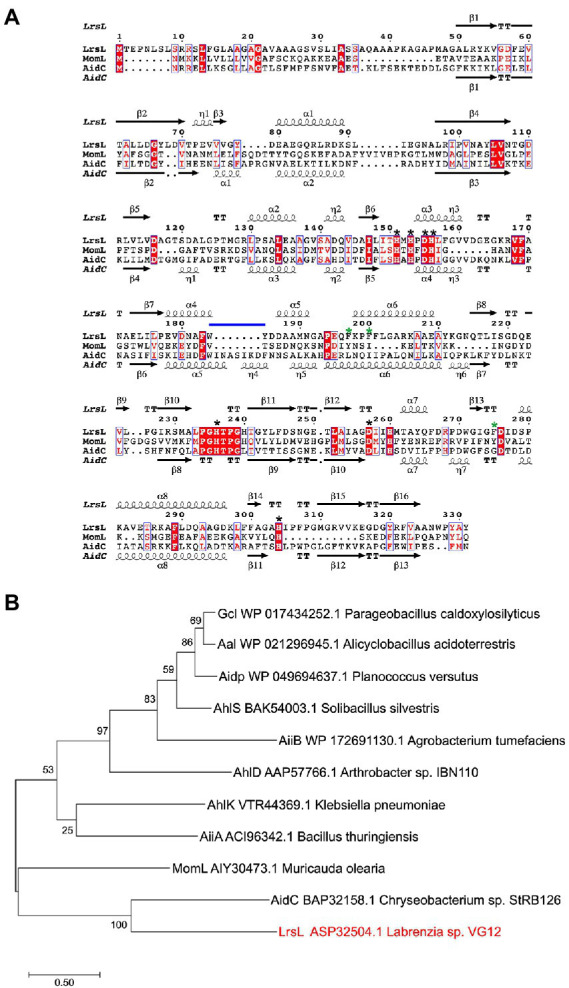
Phylogenetic analysis of QQ enzymes **(A)** annotated sequence alignment of LrsL, MomL and AidC prepared by Espript 3.0 (http://espript.ibcp.fr). Secondary structure of LrsL is shown above the sequence, and for AidC below. Conserved zinc coordination residues are marked with black asterisks. The three substrate-coordinating phenylalanines, found in LrsL, are marked with green asterisks. The atypical extension of the AidC loop between α5 and α6 is with a horizontal blue line. **(B)** Phylogenetic analysis of LrsL. A phylogenetic tree of LrsL and 10 other QQ enzymes was constructed using the Maximum Likelihood method. The end of each branch shows the name of the QQ enzyme, followed by the GenBank accession number and the name of the host bacteria. Branch node numbers represent bootstrap values and consensus inferred from 500 iterations.

We established a multiple sequence alignment of LrsL and other well-studied lactonases (Figure 1A; Supplementary Figure S2). These include AhlS (BAK54003.1) from *Solibacillus silvestris* (Morohoshi et al., 2012), AidC (BAP32158.1) from *Chryseobacterium* sp. StRB126 (Wang et al., 2012), AhlD (AAP57766.1) from *Arthrobacter* sp. IBN110 (Park et al., 2003), AidP (WP_049694637.1) from Antarctic *Planococcus versutus* (See-Too et al., 2018), AhlK (VTR44369.1) from *Klebsiella* sp. (Chan, 2013), MomL (AIY30473.1) from *Muricauda olearia* (Tang et al., 2015), Aal (WP_021296945.1) from *Alicyclobacillus acidoterrestris* (Bergonzi et al., 2018), Gcl (WP017434252.1) from *Parageobacillus caldoxylosilyticus* (Bergonzi et al., 2016), AiiA (ACI96342.1) from *Bacillus thuringiensis* (Liu et al., 2005), and AiiB (WP_172691130.1) from *Agrobacterium tumefaciens* (Liu et al., 2007). The AiiA sequence in the NCBI database was truncated. We added 19 amino acids at the N-terminal to obtain the sequence given by (Liu et al., 2007). The HXHXDH~H (where X is any residue and ~ indicates a long separation) motif is considered the hallmark of MBL superfamily (Wang et al., 2012). All lactonases included shared the HXHXDH~H motif confirming they belong to the MBL superfamily (Figure 1A; Supplementary Figure S2; Dong et al., 2000; Wang et al., 2012). This motif is involved in metal ion coordination and is essential for the function of lactonases (Dong et al., 2002; Palzkill, 2013). AhlK carries an asparagine residue instead of a second aspartic acid at position 257 (Supplementary Figure S2, blue asterisk). The multiple sequence alignment demonstrated that His260 (numbering scheme corresponding to wild-type LrsL) was conserved in AidC, MomL, and LrsL. In contrast, AiiA, AiiB, Aal, Gcl, AhlK, AhlD, AhlS, and AidP all carry a conserved tyrosine in this position (Supplementary Figure S2, black asterisk). This tyrosine in AiiA and histidine in MomL are required for substrate binding and catalysis (Momb et al., 2008; Tang et al., 2015).

**Figure 2:**
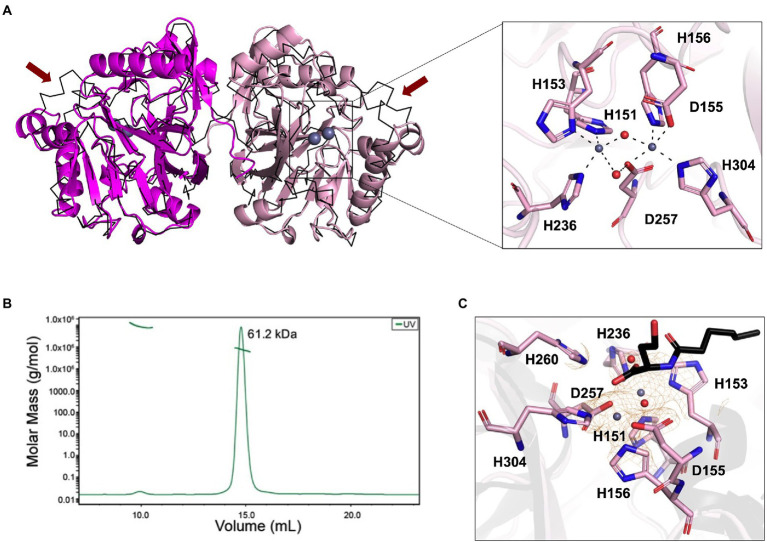
Structural analysis of LrsL. **(A)** Dimeric crystal structure of LrsL. (*Left*) The structure of LrsL (residue 49 to C-terminal) is shown as cartoon diagram, with one chain in magenta and the other chain in light pink. The C-alpha trace (black) of the experimental structure of the AidC dimer (PDB accession 4zo3) is superimposed. The two zinc ions included in each LrsL protein chain are shown in blue-gray. (*Right*) Zoom into the zinc-binding active site binding pocket of LrsL. The zinc-coordinating residues as highlighted in Figure 1A and Supplementary Figure S2 are marked as light pink sticks. Zinc ions are shown as blue-gray spheres. Zinc coordinating atoms are indicated by dashed lines from the atom to zinc. The red spheres indicate water molecules that contribute to zinc coordination in LrsL. **(B)** Size Exclusion Chromatography coupled with Multiangle Light Scattering (SEC-MALS) analysis showed that LrsL eluted as a single peak with a molecular weight of 60.2 kDa, corresponding to the molecular weight of an LrsL dimer. **(C)** 2*F*_o_-*F*_c_ map (orange mesh) for the unliganded LrsL zinc ions and water molecules. The map is contoured at 2σ. The LrsL zinc-binding site is superimposed on the crystal structure of AidC:C6L complex (PDB 4zo3), showing electron density close to the C6L O1 atom in the AidC-C6L complex structure (C6L is shown as a stick model with carbon atoms in black). Residue coloring corresponds to the right panel in **(A)**.

The phylogenetic analysis revealed that the newly isolated LrsL was closely related to AidC, which forms stable zinc-binding homodimers (Figure 1B; Mascarenhas et al., 2015). The MBL superfamily contains monomeric, dimeric (Mascarenhas et al., 2015), and tetrameric enzymes (Ullah et al., 1998; Mercuri et al., 2001; Li de la Sierra-Gallay et al., 2005). To gain experimental insights into the mechanism of catalysis and self-assembly, we determined the three-dimensional structure of LrsL, showing residues 49–331 at a resolution of 1.9 Å (Figure 2A; Table 2). The crystals contain a symmetric LrsL dimer in the asymmetric unit, burying a total protein surface of 1,135 Å^2^ in the interface. Size exclusion chromatography–multiangle light scattering analysis confirmed that LrsL was eluted as a single peak with a molecular mass of 61.2 kDa corresponding to an LrsL dimer (Figure 2B). The LrsL dimeric arrangement resembles closely the one of AidC, with an r.m.s.d of 0.952 Å (Figure 2A). However, AidC has an extension of the loop between helices α5 and α6, which is absent in LrsL and most other lactonases (Figures 1A, 2A; Supplementary Figure S2). This loop extension covers one side of the substrate binding site and narrows substrate selectivity in AidC (Mascarenhas et al., 2015).

**Table 2:** X-ray diffraction data collection and refinement statistics for LrsL.

	LrsL
Wavelength (Å)	0.987
Resolution range	46.18–1.89 (1.958–1.89)
Space group	P 41 3 2
Unit cell	166.51 166.51 166.51 90 90 90
Total reflections	5,147,810 (520,169)
Unique reflections	63,335 (6,220)
Multiplicity	81.3 (83.6)
Completeness (%)	99.97 (99.98)
Mean I/sigma(I)	31.63 (0.71)
Wilson B-factor	44.72
R-merge	0.1542 (6.561)
R-meas	0.1552 (6.601)
R-pim	0.01717 (0.7185)
CC1/2	1 (0.368)
CC*	1 (0.734)
Reflections used in refinement	63,327 (6,219)
Reflections used for R-free	1,992 (195)
R-work	0.1902 (0.3515)
R-free	0.2290 (0.3834)
CC(work)	0.965 (0.579)
CC(free)	0.951 (0.526)
Nr. of non-hydrogen atoms	4,713
macromolecules	4,416
Ligands	4
Solvent	293
Protein residues	576
RMS(bonds)	0.011
RMS(angles)	1.81
Ramachandran favored (%)	96.68
Ramachandran allowed (%)	2.97
Ramachandran outliers (%)	0.35
Rotamer outliers (%)	4.97
Clashscore	4.95
Average B-factor	47.60
macromolecules	47.39
ligands	39.70
solvent	59.31

Statistics for the highest-resolution shell are shown in parentheses.

The crystal structure of LrsL exhibits a functional catalytic site where the conserved H^151^XH^153^XD^155^H^156^ ~ H^236^ ~ D^257^ ~ H^304^ (superscript show the position of amino acid in full-length protein) motif residues and one water molecule are coordinating two zinc ions as the metal cofactor, akin to AidC and other previously investigated lactonases (Dong et al., 2000; Figure 2A; Supplementary Figure S2). We crystallized LrsL without AHL but noticed an unoccupied electron density above the zinc molecules. This density, which we tentatively modeled as a water molecule, is in hydrogen-bonding distance to H260, and located in the same position as the AHL oxygen O1 of the AidC/N-hexanoyl-L-homoserine (C6L) complex (PDB id 4zo3; Figure 2C).

The crystal structure of LrsL was highly similar to the model predicted by AlphaFold2 (Jumper et al., 2021; r.m.s.d is 0.501 Å). Most residues, including those important for zinc binding, had been correctly placed by AlphaFold2 (Supplementary Figure S3). Minor differences were found in the side chain orientations of the phenylalanines F197, F200, and F275, and in the orientation of the helix carrying these residues. These regions have an elevated B-factor in the crystal structure, and hence the differences with the AlphaFold2 model may reflect this flexibility which could be related to catalysis/substrate binding.

### LrsL robustly degrades C6-AHLs after heat treatment

**Figure 3:**
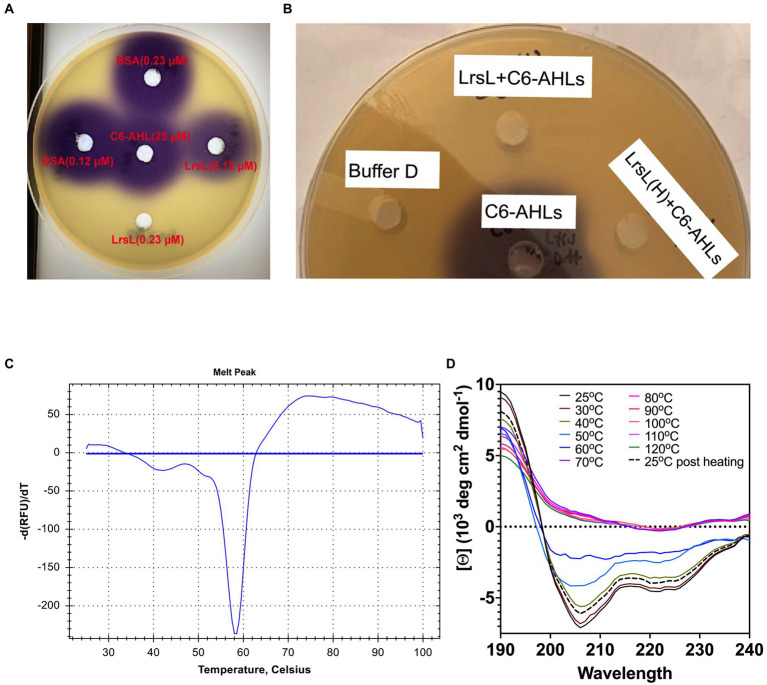
LrsL can degrade AHLs. **(A)** Two different concentrations (0.12 μM and 0.23 μM) of LrsL were incubated with 25 μM of C6-AHLs. The lower concentration (0.12 μM) of LrsL was not able to completely degrade AHLs; however, the higher concentration (0.23 μM) completely degraded AHLs. **(B)** LrsL degradation of AHL molecules. Affinity purified LrsL were incubated with 10 μM of C6-AHLs for 24 h, and the mixture was poured in wells of agar plate overlaid with *Chromobacterium violaceum* CV026. C6-AHLs in buffer D without LrsL was used as a positive control (middle well) and buffer D was used as a negative control. LrsL + C6AHLs indicates C6-AHLs treated with LrsL, while LrsL(H) + C6AHLs indicates C6-AHLs treated with heat-treated (110°C for 30 min) LrsL. The purple color development in the middle well (C6-AHLs) indicates intact AHLs, while no color indicates degradation or the absence of AHLs. **(C)** Differential Scanning Fluorimetry (DSF) thermal stability assay showing first derivative of melting curve. **(D)** Overlaid Circular Dichroism (CD) spectra of LrsL measured at temperatures from 25°C to 120°C (labeled appropriately by solid lines). The sample was cooled overnight and re-measured at 25°C (shown by a black dashed line).

Next, we investigated the ability of recombinant LrsL to degrade AHL molecules (Figure 3A). The incubation of affinity chromatography–purified LrsL with 10 μM C6-AHLs completely degraded these QS compounds, as determined using a *C. violaceum* CV026 biosensor assay on agar plates (Figure 3B). BSA was used as a control and did not degrade AHLs. The minimum active concentration of LrsL that could fully degrade 25 μM C6-AHLs in the biosensor assay was 0.23 μM (Figure 3A). This concentration is dependent on various factors, most importantly enzyme purity. The assay was performed using relatively low-purity LrsL (Figure 3A, second lane); hence, the obtained concentration should be considered an upper bound. Surprisingly, LrsL degraded C6-AHLs even after being heated in a dry bath at 110°C for 30 min, followed by cooling to room temperature and catalytic assaying. Highly thermostable lactonases, some showing a melting temperature above 100°C, have previously been reported for the PLL and MBL superfamilies, including the marine-derived MBL lactonase Aii20J (Merone et al., 2005; Hawwa et al., 2009; Cao et al., 2012; Hiblot et al., 2015; Mayer et al., 2015; Morohoshi et al., 2015; Bergonzi et al., 2018). However, *Labrenzia* was isolated from a mesophilic environment. Indeed, our thermal stability assay revealed that LrsL has a melting temperature *T_m_* of only 58.3°C (Figure 3B). Circular dichroism spectrometry confirmed that LrsL was completely unfolded at 110°C but recovered its natural fold almost completely after cooling overnight. Hence, the capability of LrsL for maintaining its catalytic activity after heat treatment is explained by its robust refolding after heat denaturation (Figure 3C).

### Kinetic parameters of LrsL outperform those of other lactonases

**Figure 4:**
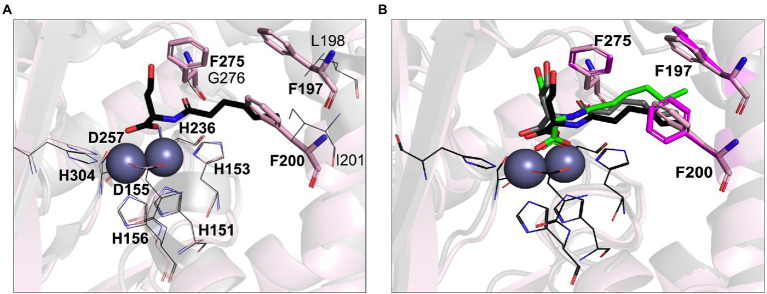
Computational AHL binding analysis. **(A)** The binding pocket of LrsL (light pink cartoon) is superimposed on AidC (PDB 4zo3; gray). The unique phenylalanine residues in the active site of LrsL are shown as light pink stick models. The corresponding AidC residues (leucine, isoleucine and glycine) are shown as dark gray lines. Conserved zinc coordination residues for LrsL (light pink lines) and AidC (black lines) are shown. Zinc ions are shown as blue-gray spheres. **(B)** Computational docking poses of C6L on AidC and LrsL. The crystallographic structure of the AidC:C6L complex is shown as a black cartoon, with C6L shown as black stick model. The redocked pose of C6L on AidC is represented as gray stick model and the docked pose of C6L on LrsL is shown as green stick model. The phenylalanine residues from the apo-crystal structure of LrsL are shown in light pink sticks, and from the C6L docked model of LrsL as magenta sticks. Conserved zinc coordination residue coloring and labeling corresponds to **(A)**. Zinc ions are shown are blue-gray spheres.

Previous studies have demonstrated that AHL lactonases exhibit *K_m_* values in the range of 7.9 μM (Gcl) to 5.6 mM (AiiA) and catalytic turnover rates (*K_cat_*) in the range of 8.9 s^−1^ (Gcl) to 91 s^−1^ (AiiA) measured against C6-AHLs (Momb et al., 2008; Bergonzi et al., 2016). Unusually low Michaelis constant *K_m_* values in the micromolar range were reported for AidC, suggesting it has a higher substrate affinity than other lactonases (Wang et al., 2012; Mascarenhas et al., 2015). Indeed, AidC ranks as one of the most efficient catalysts in terms of *K_cat_*/*K_m_* values (Mascarenhas et al., 2015). The phylogenetic proximity to AidC suggested that LrsL is also an efficient catalyst for AHL degradation. However, there are also two important structural differences between AidC and LrsL, as revealed by the crystallographic LrsL structure, which may affect the catalytic efficiency and substrate selectivity. Firstly, the atypical α5 and α6 loop extension of AidC is in a position where it interferes with AHL chains beyond carbon C5, explaining the marked influence of AHL chain lengths on catalytic activity described for AidC (Mascarenhas et al., 2015). This AidC-specific loop extension is absent in LrsL. Therefore, we expected the catalytic activity of LrsL to be relatively independent of chain lengths. Secondly, LrsL shows a unique presence of three phenylalanines (F197, F200, and F275) in a position where they can coordinate the AHL tail carbons C1–C5 particularly well, suggesting a strong interaction with the substrate (Figures 1A, 4A; Supplementary Figure S2).

To computationally explore the effect of the LrsL structural framework on substrate binding, we carried out *in silico* docking analyses. As a control and comparison, we used the crystallographic structure of AidC that had been determined with C6L (N-hexanoyl-L-homoserine) bound. Our docking procedure positioned C6L in the correct way onto AidC, validating our *in silico* approach (Figure 4B). C6L was computationally docked into the LrsL binding pocket in the same position, with the three phenylalanines engaging hydrophobic interactions with the carbon tail (Figure 4B). The calculated energy gain for C6L binding to LrsL was −9.1 kcal/mol, which was significantly larger than the −7.4 kcal/mol calculated for the (redocked) C6L binding to AidC. This analysis suggested that LrsL has an even higher affinity for the AHLs than AidC, and hence an even lower *K_m_*.

To experimentally assess the structure-based prediction that LrsL is an extremely efficient catalyst for AHLs with a range of tail lengths, we established the catalytic parameters of LrsL for three AHLs: C4-AHLs, C6-AHLs, and oxo-C12-AHLs (Table 3; Supplementary Figure S4). The C4-AHLs and oxo-C12-AHLs are used by the opportunistic pathogen *P. aeruginosa* to regulate virulence and biofilm formation. Indeed, LrsL exhibited a low *K_m_* of only 6 μM for C6-AHLs (Table 3). This *K_m_* value was markedly lower compared with other well-studied lactonases for C6-AHLs, such as AiiA and AiiB (*K_m_* = 1.6 mM and 5.6 mM, respectively; Liu et al., 2007; Momb et al., 2008), and with marine-derived MomL (*K_m_* = 790 μM; Tang et al., 2015). Further, LrsL also exhibited lower *K_m_* values than AidC (*K_m_* = 61 μM; Mascarenhas et al., 2015). Therefore, the substrate affinity of LrsL for C6-AHLs is higher than the substrate affinities of other previously reported AHL lactonases. As predicted, LrsL catalyzed C4-AHLs and oxo-C12-AHLs with *K_m_* values very similar to C6-AHLs (Table 3). The *K_m_* values are a limiting factor for lactonases, as the concentration of AHLs in natural and environmental settings usually ranges from pico to nanomoles per liter (Tan et al., 2014; Dal Bello et al., 2021). In biotech applications, lactonases with the lowest *K_m_* values are desirable, because they efficiently bind and degrade AHLs at lower concentrations, limiting the ability of bacteria to communicate.

**Table 3:** Kinetic parameters of LrsL evaluated at 25°C.

AHLs	*K_cat_* (s^−1^)	*K_m_* (μM)	*K_cat_*/*K_m_* (s^−1^ M^−1^)
C4-AHLs	101.3 ± 3.06	3 ± 0.88	(3. 37 ± 0.99) × 10^7^
C6-AHLs	137.3 ± 6.3	6 ± 2.0	(2. 28 ± 0.26) × 10^7^
Oxo-C12-AHLs	124.0 ± 4.25	5 ± 0.95	(2. 48 ± 0.48) × 10^7^

Values are given with ± standard deviation derived from three independent experiments.

Accordingly, the combination of a faster hydrolysis rate (*K_cat_*) and higher substrate affinities (*K_m_*) in LrsL resulted in a catalytic efficiency (*k_cat_*/*K_m_* = 3 × 10^7^ M^−1^) for LrsL that was substantially higher than for lactonases previously considered the most efficient catalysts (Table 3). For example, Gcl and AidC lactonases have catalytic efficiencies for C6-AHLs of 1.12 × 10^6^ M^−1^ s^−1^ and 9.7 × 10^5^ M^−1^ s^−1^, respectively (Mascarenhas et al., 2015; Bergonzi et al., 2016). Hence, based on the catalytic efficiency (*k_cat_*/*K_m_*), LrsL is currently the most efficient QQ enzyme available.

### LrsL effectively inhibits biofilm formation

**Figure 5:**
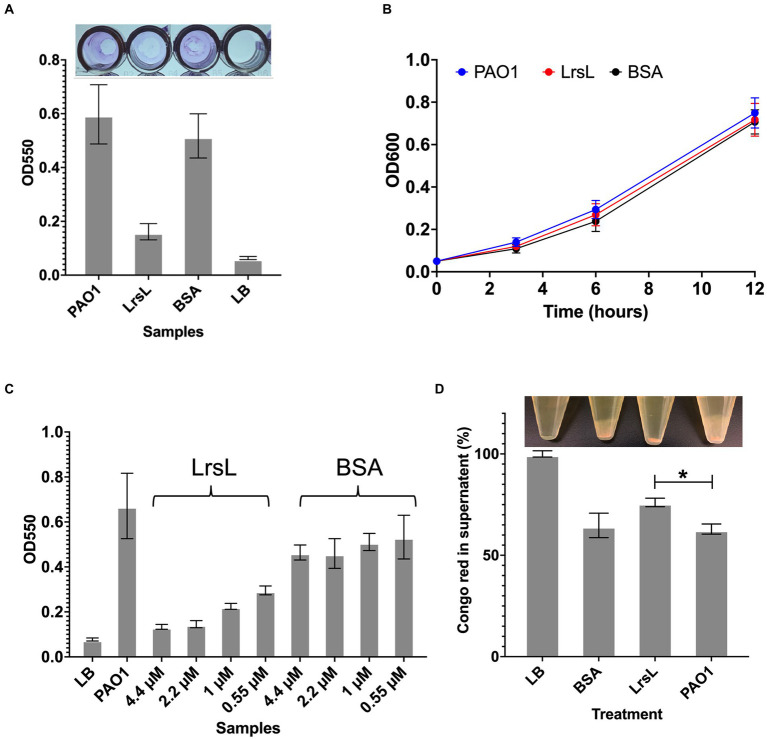
LrsL reduces biofilm formation. **(A)** The upper panel shows the photo of wells with biofilm formed by *Pseudomonas aeruginosa* PAO1 and various treatments. The lower panel shows the corresponding quantification of biofilms. The *y*-axis shows absorbance at 550 nm, and the *x*-axis shows biofilm formed by PAO1(with buffer D) and PAO1 with LrsL, and BSA. Unpaired student’s *t*-test was applied to measure the significance; value of *p* < 0.001 are indicated by asterisks. **(B)** The growth curves of *P. aeruginosa* (PAO1) and PAO1 treated with LrsL and BSA shows no significant difference. **(C)** LrsL reduces biofilm formation in a dose-dependent manner. Increasing the dose of LrsL increases the suppression of biofilm formation by *P. aeruginosa*. The *y*-axis shows absorbance at 550 nm, and the *x*-axis shows biofilm formed by PAO1 (with buffer D) and PAO1 treated with various concentrations of LrsL and BSA. The LrsL concentration was increased from 0.55 μM to 4.43 μM. As a control, PAO1 was also incubated with similar concentrations of BSA. For all the experiments, LB was used as a negative control. Error bars indicate the standard deviation of three biological replicates (eight wells each). **(D)** LrsL interferes with Pel production by *P. aeruginosa*. The upper panel show photographs of blank (LB) and *P. aeruginosa* cultures incubated with BSA, LrsL, and buffer D (PAO1). The lower panel shows the quantification of Pel using the Congo Red (CR) binding assay. For LB, all the CR remained in the supernatant, represented as 100%. In the presence of LrsL, Pel production was compromised, and more CR remained in supernatant compared to BSA and PAO1. The difference between CR in the supernatant of LrsL-treated and untreated PAO1 was significantly higher as measured by the student’s *t*-test, shown by an asterisk (value of *p* = 0.005). Error bars represent the standard deviation of three biological replicates.

Next, we investigated the functional effects of the high *in vitro* catalytic rates of LrsL. We assessed the ability of LrsL to inhibit biofilm formation by *P. aeruginosa* PAO1, an important opportunistic pathogen commonly responsible for nosocomial infections. QS plays a vital role in biofilm formation by *P. aeruginosa* (Davies et al., 1998; Schuster et al., 2013), and an interference with its production of AHLs leads to the inhibition or formation of defective biofilms (Davies et al., 1998; Shih and Huang, 2002). Recently, it was confirmed that QQ enzymes reduce the biomass and density of biofilms formed by *P. aeruginosa* (Malesevic et al., 2020). We found that the application of 1.14 μM LrsL reduced the biofilm formation by more than 3-fold compared to untreated cultures and to cultures treated with BSA (Figure 5A). Similar to previous reports, we found no negative effect of LrsL on the growth of bacteria (Figure 5B; Bergonzi et al., 2018, 2019), further supporting that the use of QQ enzymes may not promote the development of resistance in bacteria.

The inhibitory effect of LrsL was dose-dependent. The application of 0.55 μM LrsL reduced the biofilm by 2.2-fold. Increasing the concentration of LrsL from 0.55 μM to 4.4 μM further reduced the biofilm by another 2.2-fold (Figure 5C). However, no dose-dependent response was observed for BSA, used as control. The treatment of an existing *P. aeruginosa* biofilm with LrsL for 6 h resulted in the decrease in the total biofilm thickness at the time of measurement by more than 50% compared with untreated or BSA-treated controls, without affecting bacterial growth (Supplementary Figure S5). It remains to be determined if the apparent biofilm decrease is solely the result of LrsL inhibiting the growth and/or renewal of the biofilm over the measurement period, or if LrsL can also disrupt preformed biofilms.

### LrsL interferes with exopolysaccharide production

Normal *P. aeruginosa* biofilm production requires the exopolysaccharides Pel and Psl (Colvin et al., 2012). Therefore, we next asked whether LrsL interferes with biofilm production by causing changes in the production of Pel and/or Psl. Congo Red (CR) is a dye known to bind to Pel and Psl (Friedman and Kolter, 2004; Ma et al., 2006). When Pel and/or Psl are produced, they bind and precipitate CR, leaving a lower amount of CR in the supernatant. Consequently, bacterial strains deficient in Pel/Psl production reduce their binding to CR. (Friedman and Kolter, 2004; Ma et al., 2006). We, therefore, monitored supernatant CR to probe the effect of LrsL on Pel/Psl production.

Following *P. aeruginosa* PAO1 treatment with purified LrsL, we observed that most of the CR (76% ± 2.1) remained in the supernatant, suggesting suppressed production of Pel or Psl. In contrast, PAO1 and PAO1 treated with BSA exhibited a significantly lower amount of CR in the supernatant (63% ± 2.5 and 64% ± 6.03, respectively), inferring Pel production (Figure 5D). The effect of LrsL on Pel/Psl production was visible after the cultures were centrifuged. The LrsL-treated PAO1 lacked cloudy substances (likely comprising Pel/Psl and other extracellular polymeric substances) over the cell pellet, in contrast to PAO1 alone and PAO1 treated with BSA (Figure 5D, top). These results indicate that the application of LrsL disrupts the production of polysaccharides. Pel is upregulated by QS, and the addition of 3-oxo-C12-AHLs to QS mutants of *P. aeruginosa* restored Pel production (Sakuragi and Kolter, 2007). However, also Psl has been linked to QS (Attila et al., 2008). Hence, the relative contribution of each to biofilm inhibition by LrsL, as well as the molecular mechanisms linking QQ to decreased exopolysaccharide production remain to be investigated.

## Conclusion

Biofilm formation or biofouling is a major problem in various fields because biofilms deteriorate materials and cause infections. Current antifouling and biofilm treatment procedures are toxic to the environment. The QQ enzymes may be a sustainable and eco-friendly class of agents that can be applied to counter biofilms and treat infections caused by pathogenic bacteria.

In this study, we characterized a novel QQ enzyme, LrsL, from the Red Sea sediment bacterium *Labrenzia* sp. VG12. Evolutionary analysis showed that LrsL belongs to the MBL superfamily whose members, such as GcL, MomL, and AiiA display broad substrate specificity (Wang et al., 2004; Mascarenhas et al., 2015; Tang et al., 2015; Bergonzi et al., 2019). Experimental and *in silico* analysis of LrsL indicated that LrsL can catalyze AHLs with varying tail lengths, and has evolved a unique hydrophobic mechanism to capture its substrates. The flexibility of the phenylalanine side chains and the absence of an AidC-like closure of the substrate binding pocket suggest that LrsL can also catalyze additional AHLs. We confirmed experimentally that LrsL is a broad spectrum QQ enzyme that degrades C4-AHLs, C6-AHLs, and 3-oxo-C12-AHLs with a catalytic efficiency of ~10^7^ M^−1^ s^−1^. To date, this catalytic efficiency is the highest reported for any QQ lactonase. Furthermore, LrsL exhibited a very high affinity for different AHLs, making it an enzyme of choice in environments with low AHL concentrations. As an enzyme secreted in an ocean environment, this characteristic may be required to capture highly diluted substrates.

We found that purified LrsL suppresses the biofilm formation by *P. aeruginosa*, a ubiquitous bacterium involved in biofilm and nosocomial infections. Therefore, LrsL may help to prevent or limit biofilm formation on susceptible surfaces, such as medical devices and those submerged in water. Although LrsL will lose its structure and function at temperatures above 50°C, its capability to refold and regain function even after prolonged heat treatment at 120°C can facilitate its clinical and industrial applications.

Our experiments open the possibility that LrsL also breaks down existing *P. aeruginosa* biofilms. If confirmed through follow-up studies, this function would allow using LrsL and possibly other QQs to increase susceptibility of biofilm-forming bacteria to antimicrobials (Kiran et al., 2011). Thus, QQ enzymes would decrease the number of antimicrobials needed, which could limit the spread of antimicrobial resistance.

The LrsL-based disruption of exopolysaccharide production by *P. aeruginosa* may also have important implications for medical applications. Although the role of Pel and Psl in *P. aeruginosa* biofilm formation is strain-specific, both play a vital role in adherence to the surface and maintaining cell–cell interaction within a biofilm (Vasseur et al., 2005; Colvin et al., 2011, 2012). Furthermore, Pel protects biofilm bacteria against certain aminoglycoside antibiotics (Colvin et al., 2011). Therefore, interference with Pel production would likely compromise the ability of *P. aeruginosa* to form biofilms and render existing biofilms more susceptible to antimicrobial treatment (Kiran et al., 2011). This outcome is especially important, as *P. aeruginosa* is inherently resistant to antimicrobials, and biofilm formation increases the amount of antimicrobial that would be required. Therefore, even if QQ enzymes cannot completely remove biofilms, they may lower the amount of antimicrobial or cleaning chemicals required to clear biofilms.

Collectively, our work supports that QQ enzymes are promising agents to control biofouling and antimicrobial resistance in the clinic and in various industries.

## Data availability statement

The datasets presented in this study can be found in online repositories. The names of the repository/repositories and accession number(s) can be found in the article/Supplementary material. Atomic coordinates for LrsL have been deposited in the Protein Data Bank (PDB) with accession code 7Y7U.

## Author contributions

ZR, AM, RG, TI, and SA designed the experiments. ZR, AM, and AA performed the experiments. AM and SA carried out X-ray crystallography and computational analyses. ZR, AM, RG, and SA wrote and revised the manuscript. All authors contributed to the article and approved the submitted version.

## Funding

This research was supported by the King Abdullah University of Science and Technology (KAUST) through the baseline fund and Award No. URF/1/1976-36-01 from the Office of Sponsored Research.

## Conflict of interest

The authors declare that the research was conducted in the absence of any commercial or financial relationships that could be construed as a potential conflict of interest.

## Publisher’s note

All claims expressed in this article are solely those of the authors and do not necessarily represent those of their affiliated organizations, or those of the publisher, the editors and the reviewers. Any product that may be evaluated in this article, or claim that may be made by its manufacturer, is not guaranteed or endorsed by the publisher.

## Footnotes

^1^ https://web.expasy.org/protparam/

^2^ www.pymol.org

^3^ www.graphpad.com
