# Supplementary material for: The exceptionally efficient quorum quenching enzyme LrsL suppresses Pseudomonas aeruginosa biofilm production
Source: Front Microbiol. 2022 Aug 22;13:977673. doi: 10.3389/fmicb.2022.977673 (PMC9441902; doi:10.3389/fmicb.2022.977673)
Supplement: Supplementary file 1 [file Data_Sheet_1.pdf]

Supplementary Material

The exceptionally efficient quorum quenching enzyme LrsL suppresses *Pseudomonas aeruginosa* biofilm production

Zahid Ur Rehman[1,2*,†], Afaque A. Momin[1,3,†], Abdullah Aldehaiman[1,3], Tayyaba Irum[4], Raik Grünberg[1,3], Stefan T. Arold[1,3,5*]

[1]Bioscience Program, Biological and Environmental Science and Engineering Division, King Abdullah University of Science and Technology (KAUST), Thuwal, Kingdom of Saudi Arabia
[2]Environmental Science Program, Water Desalination and Reuse Center, King Abdullah University of Science and Technology (KAUST), Thuwal, Kingdom of Saudi Arabia
[3]Computational Biology Research Center, King Abdullah University of Science and Technology (KAUST), Thuwal, Kingdom of Saudi Arabia
[4]Services Hospital, Services Institute of Medical Sciences, Lahore, Pakistan
[5]Centre de Biologie Structurale (CBS), INSERM, CNRS, Université de Montpellier, F-34090 Montpellier, France

[†]These authors have contributed equally to this work.

*Correspondence:

Tel. +966 12 808 0192; Email: zahid.urrehman@kaust.edu.sa

Tel. +966 12 808 2557; Email: stefan.arold@kaust.edu.sa

Figure S1: (A) Purification of LrsL using chromatography. The first lane shows a ladder with molecular weight given in kDa. The second, third, and third lane shows LrsL purified using affinity (Twin Strep-tag), Ion-Exchange (IEX), and Size Exclusion Chromatography (SEC). An arrow indicates the 35 kDa (with Twin-strep tag) LrsL. **(B)** LrsL carries an N-terminal TAT secretion signal. SignalP analysis shows a high probability of TAT (Twin-arginine translocation) signal at N-terminal with probable cleavage site at Alanine residue at 38th position.

A

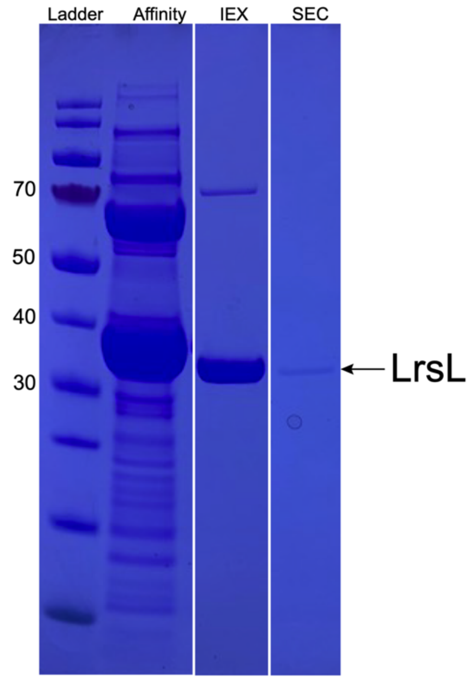

B

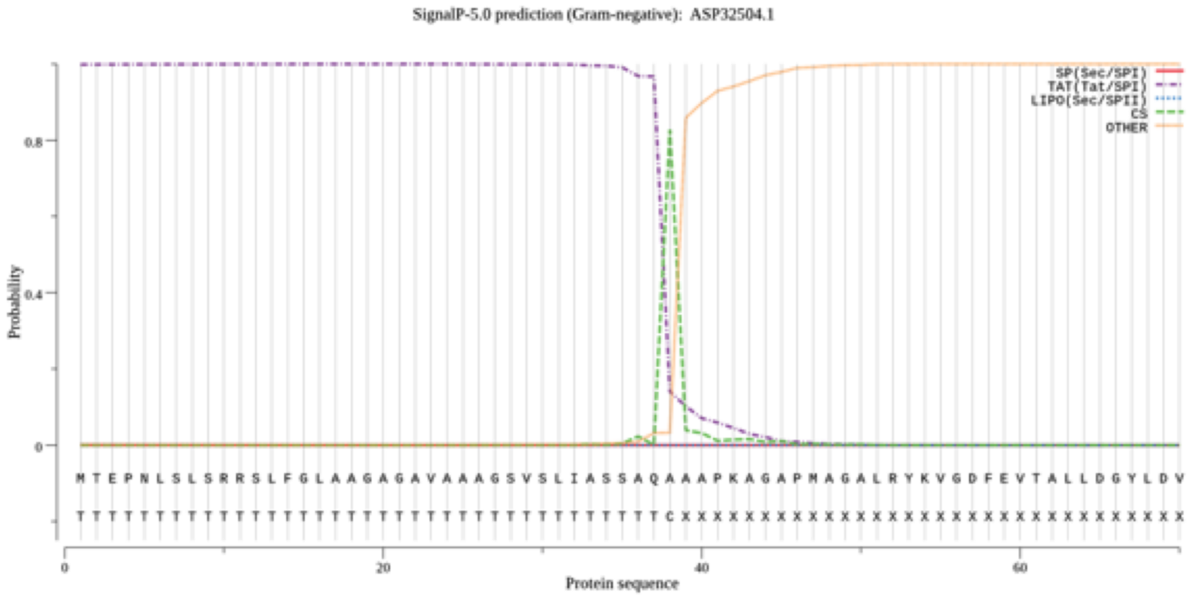

Figure S2: Multiple Sequence Alignment of LrsL with other MBL lactonases. The LrsL was aligned to other functionally characterized lactonases. The HXHXDH~H motif involved in metal coordination is given in purple boxes (Kim et al., 2005). Amino acids conservation and consensus panels are given below the alignment. The blue and black asterisks points to the residues involved in catalytic activity, and the red asterisks indicate the positions of the phenylalanine residues in LrsL surrounding the binding pocket.

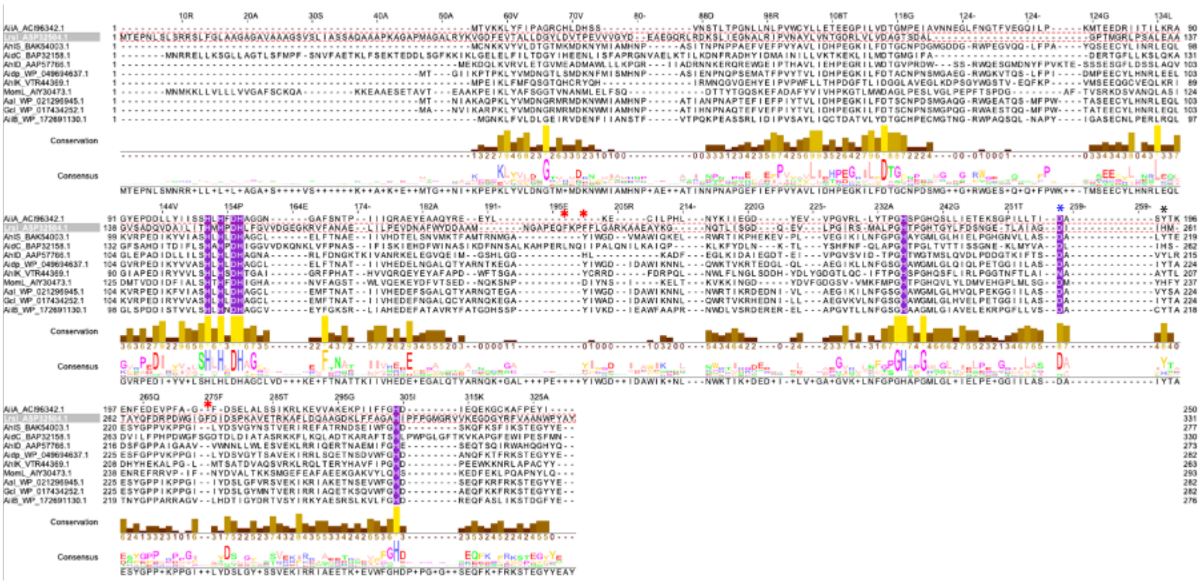

Figure S3: ***(Left)*** Dimeric crystal structure of LrsL (light pink) superimposed on the AlphaFold predicted model (cyan), both shown as cartoon diagrams. ***(Right)*** Zoom into the zinc-binding active site of LrsL. The zinc-coordinating residues are highlighted as lines, and the phenylalanines surrounding the binding site are highlighted as sticks. Zinc ions are shown as blue-gray spheres.

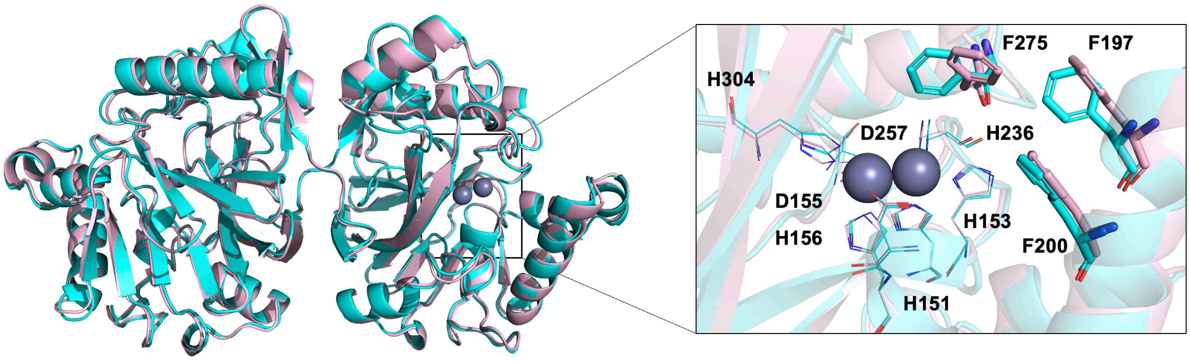

Figure S4: Hyperbolic curve fitting model for calculating LrsL catalytic parameters against three AHLs (C4, C6, and Oxo-C12). The y-axis shows initial velocities and x-axis show substrate concentration of C4-AHLs, C6-AHLs, Oxo-C12-AHLs.

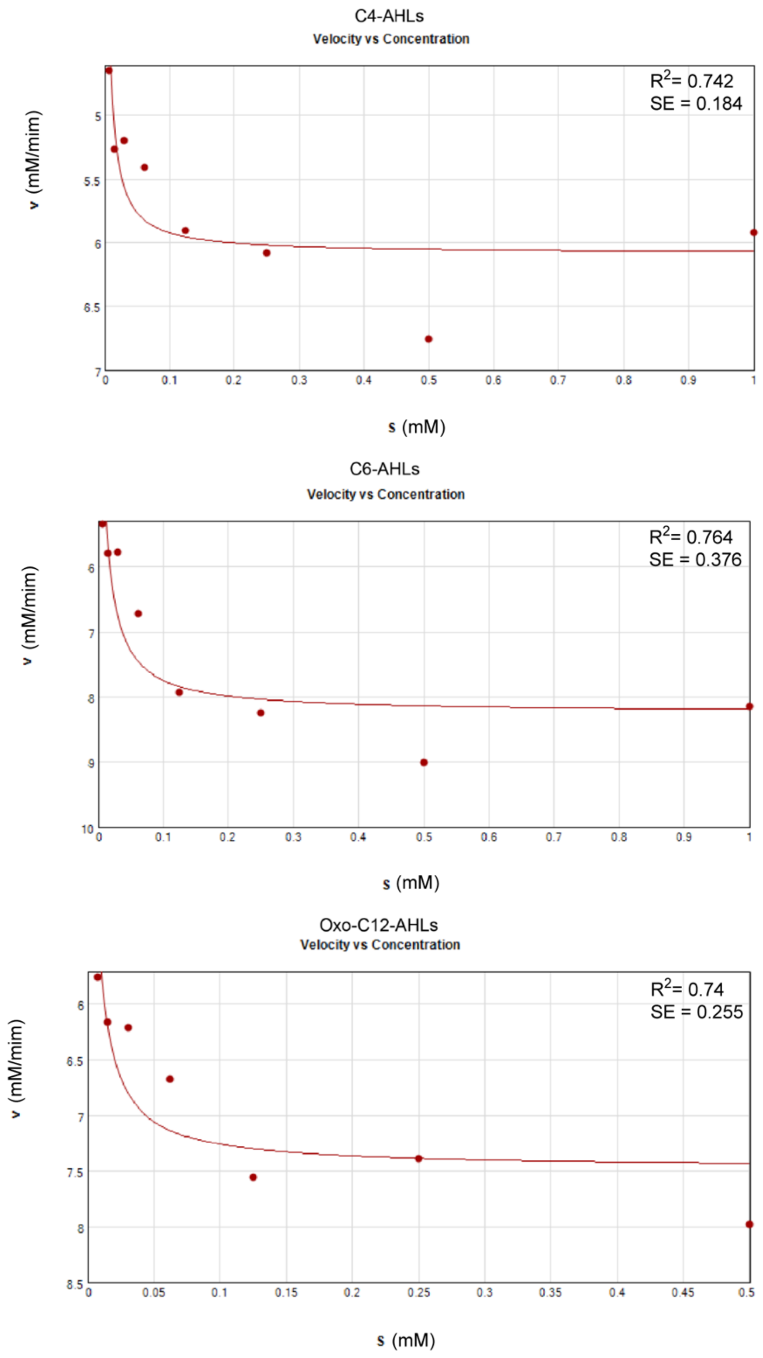

Figure S5: LrsL removes pre-formed biofilms. Preformed *P. aeruginosa* biofilms were incubated with LrsL and BSA (both in buffer D) or sterile buffer D (PAO1). LB and BSA were used as control. The y-axis shows absorbance at 550 nm. The x-axis indicates various treatments of *P. aeruginosa* biofilm.

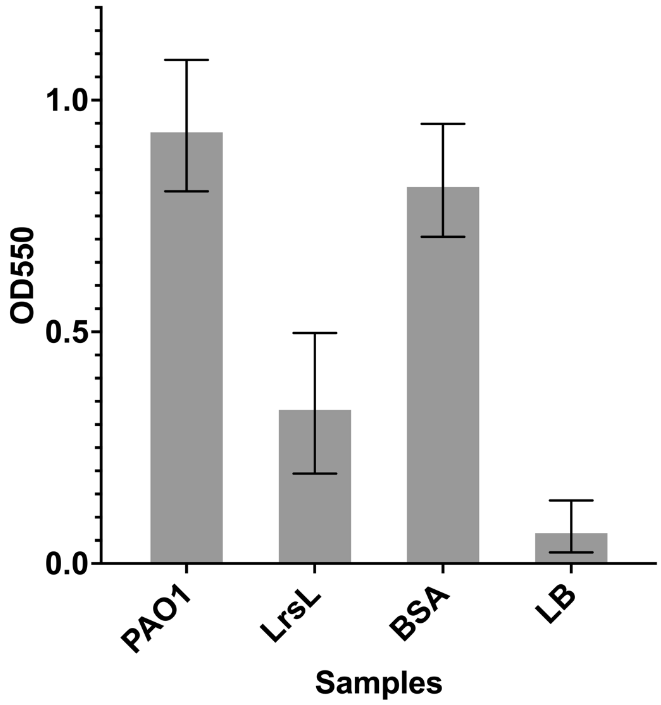

References

Kim, M.H., Choi, W.C., Kang, H.O., Lee, J.S., Kang, B.S., Kim, K.J., Derewenda, Z.S., Oh, T.K., Lee, C.H. and Lee, J.K. 2005. The molecular structure and catalytic mechanism of a quorum-quenching N-acyl-L-homoserine lactone hydrolase. P Natl Acad Sci USA 102(49), 17606-17611.
